# Prevalence of cardiorenal syndrome in type 2 diabetic patients

**DOI:** 10.6026/973206300222989

**Published:** 2026-05-31

**Authors:** Shivam Chaudhary, Bhanpratap Ahirwar, Vinay Verma, Sachin Parmar

**Affiliations:** 1 Department of General Medicine, V.K.S. Government Medical College, Neemuch, Madhya Pradesh, India; 2 Department of Community Medicine Department, V.K.S. Government Medical College, Neemuch, Madhya Pradesh, India

**Keywords:** Cardio renal syndrome, Type 2 diabetes mellitus, LVEF

## Abstract

Cardio renal syndrome (CRS) is a two-way heart-kidney disease and Type 2 Diabetes Mellitus (T2DM) is one of the major predisposing factors. CRS was found in 37.78 percent of patients in this 18-month hospital-based investigation of T2DM patients at Chirayu Medical College & Hospital, Bhopal, with type 4 CRS predominating (15.56) followed by type 2 (11.11) and type 1 (6.67) CRS. CRS patients had a major decrease in left ventricular ejection fraction and worse renal biomarkers (serum urea, egger, serum cretonne), and no difference between patients with and without CRS on HbA1c and T2DM duration. These results endorse regular echocardiographic and renal dysfunction evaluation among T2DM patients are needed to identify CRS in time and provide treatment.

## Background:

T2DM is rapidly growing diabetes pandemic affecting millions worldwide. T2DM affected 506 million people in 2021, with 23.9 million new cases and 1.6 million deaths [1]. T2DM's systemic complications, which affect the cardiovascular, renal, and glycemic systems, increase its burden [2]. Adult T2DM patients have 6 times and 2.6 times the heart disease morbidity and mortality of non-diabetics. Diabetic CKD dominates kidney failure worldwide [3]. Cardio renal syndrome (CRS) is a two-way dysfunction of the heart and kidney in type 2 diabetes mellitus (T2DM) when it has cardiac and renal impairment [4]. Rancho *et al.* explain that there are five types of CRS, with the first and second being the result of the kidney damage caused by acute and chronic heart dysfunction; the third and fourth results of the acute and chronic kidney disease with heart problems; the fifth type is the products of the systemic illnesses such as T2DM or sepsis, which harms both organs [5]. As it is observed by Rangaswami *et al.* CRS pathophysiology in T2DM is the product of interactions with hemodynamic, neurohormonal and inflammatory pathways [6]. Renal activation by the RAAS causes oxidative stress, uremic toxin load, and kidney fibrosis, which injures the heart and kidneys [7]. High blood sugar, reactive oxygen species, advanced glycation end-products, and lipotoxicity cause diabetic cardiomyopathy in T2DM patients [8]. In heart failure patients with CKD, cardiorenal interactions worsen and all-cause mortality raises 2-3-fold [9]. A decrease in eGFR and an increase in creatinine are signs of progressive nephron loss due to dysfunctional cardiac output and venous congestion [10]. CRS in hospitalized patients is rare and varies across studies. Prothasis *et al.* [11] found 17.5% internal medicine ward hospitalizations, mostly in men. Maisons *et al.* [2] found that T2DM doubled the frequency of CRS in a French national cohort, with 43% of CRS patients having T2DM. There is little research on CRS in Indian T2DM patients, despite its growing prevalence. Early diagnosis and targeted treatment require more understanding of CRS subtype trends, clinical variables, and organ-specific biomarkers in this population. Therefore, it is of interest to study the prevalence and distribution of CRS in tertiary care hospitalized T2DM patients.

## Materials and Methods:

The study was carried out at the Department of General Medicine, Chirayu Medical College and Hospital, Bhopal, Madhya Pradesh, India, in an 18 months’ timeframe (18 months between August 2022 to January 2024) after obtaining the consent of the Institutional Ethics Committee. To establish a sample size of 45, the absolute precision of 5% was used and the confidence level was 95%. Both male and female patients aged over 40 years with confirmed Type 2 Diabetes Mellitus (T2DM) were included, whereas the patients with Type 1 DM, gestational diabetes mellitus (GDM), and secondary diabetes, patients with sepsis, amyloidosis, systemic lupus erythematous (SLE), vacuities, hypertension, or hepatic cirrhosis were excluded, and those suspected, a possible patient of non-diabetic nephropathy (contracted or cystic kidney All patients who were enrolled to the study signed informed consent in their native language and written informed consent was given after being made aware of the study intention. Following a detailed history of Non-Insulin Dependent Diabetes Mellitus (NIDDM) and comorbid cardiac and renal abnormalities were obtained, a thorough general and systemic examination was conducted on every patient. Relevant investigations were done to all the subjects, such as random blood sugar (RBS), fasting plasma glucose, post prandial plasma glucose, glycine hemoglobin (HbA1c), urine routine and microscopy, renal function tests (serum urea, serum cretonne, and eGFR), urine protein-cretonne ratio (UPCR), ultrasonography of kidney-ureter-bladder (USG KUB), electrocardiogram (ECG) and two-dimensional ech The classification of cardiorenal syndrome was Type 1-5 according to the classification proposed by Ronco *et al.* [5] All the data were entered into a form of predetermined preformats and then were compiled in Microsoft Excel, statistical analysis was done using version 20.0 of SPSS and version 19.5 of MedCalc and a p-value of less than 0.05 was considered significant.

## Results and Discussion:

This study investigated cardiorenal syndrome (CRS) prevalence, its types, and clinical determinants in a patient cohort of T2DM admitted to a tertiary care unit. In our study, the total prevalence of CRS was 37.78% (Table 1). This aligns with the general body of literature that identifies T2DM as a significant risk factor of CRS. The study of Maisons *et al.* [2] in a massive French national hospital cohort of more than five million patients has shown that T2DM increased the risk of CRS twofold (adjusted HR 2.14; 95% CI 2.10-2.19), and that in patients with CRS, 43% also had T2DM. The incidence rate was higher in our study than in Maisons *et al.* [2] (around 8.5%/per year in patients with T2DM), probably due to the different study design - we specifically targeted patients presenting with both cardiac and renal complaints, which is enriched with CRS. According to an observational study of CRS types by Prothasis, Maria *et al.* [12], the cumulative hospital prevalence was 17.5% in an internal medicine ward with a cumulative prevalence of CRS higher in males (68.9%). Conversely, our research identified a greater proportional CRS burden amongst females (50 percent of enrolled female patients), which should be further investigated in more substantial prospective cohorts. The most prevalent CRS Type 4 in our cohort was chronic kidney disease with cardiac dysfunction (15.56) (Table 1). It is consistent with the results of H, Suresh *et al.* [12], who noted a high prevalence of CRS Type 4 up to 76.25% in patients with established CKD and a high cardiac presentation manifested by left ventricular failure. Our prevalence is less, but this can be explained by the mixed (T2DM-oriented) cohort under study, instead of a population exclusively with CKD. All of our patients with CRS-4 were confirmed CKD by both biochemical and ultrasonographic criteria, and CMD Lost was the consistent USG result in our study (Table 5). The StatPearls chapter describes CRS type 4 as chronic renocardiac syndrome, where established CKD leads over time to structural and functional cardiac disease, including heart failure [13]. Corticomedullary differentiation loss on USG is an established sign of underlying chronic renal parenchymal disease and is associated with low cortical thickness and poor eGFR, as indicated by Moghazi *et al.* [14] who reported a statistically significant correlation between cortical thickness on USG and eGFR (p<0.0001).Our analysis revealed that even though the total number of patients was more numerous among men (60%), CRS was disproportionately more common among women (Table 2). In a review of the CARDIOREN Spanish registry of 1107 chronic ambulatory heart failure patients, Gomez-Otero *et al.* [15] observed that advanced CKD with congestion and HFpEF (a cardiorenal phenotype) was relatively common in women, compared with HFrEF and ischemic cardiomyopathy that were common in men. This male-female trend aligns with the pattern in our data, where females were disproportionately affected by CRS, even though they were less represented in the cohort as a whole. A statistically significant result of this study was the significant difference in LVEF between CRS-present and CRS-absent patients (35.18% vs. 46.96; p<0.0001) (Table 3). Reduced LVEF indicates systolic impairment, a known cause of decreased renal perfusion and the subsequent cardiorenal pathophysiology. A review of the literature on cardiorenal syndrome due to heart failure with preserved ejection fraction by Ahmed and Campbell [16] noted the co-occurrence of renal impairment, a condition that is prevalent in older women, and that significantly deteriorates prognosis. The LVEF distinction was highly significant in our study, and this underlines the importance of 2D echocardiography as a non-invasive, indispensable tool in risk stratification of CRS in T2DM patients.CRS patients in our cohort exhibited highly deranged renal biomarkers - eGFR 27.47 ml/min/1.73m2 (Stage 3b-4 CKD), creatinine 2.58 mg/dl, and urea 60.12 mg/dl (Table 3) - that were significantly above non-CRS patients (p<0.0001 of all three). These data are consistent with the fact that the cycle of CRS worsens progressive kidney malfunction through decreased cardiac output, neurohormonal stimulation, and venous stasis. The scientific statement by Ronco *et al.* [5] of the American Heart Association characterized eGFR loss and creatinine increase as defining cardinal biomarkers delineating the cardiorenal continuum. Our CRS patients are at a high risk of developing end-stage renal disease due to dramatically depressed eGFR (less than 30 ml/min/1.73m2). However, surprisingly, there was no significant difference in HbA1c between CRS and non-CRS groups (8.23% vs. 8.57%; p=0.4371), or duration of T2DM (8.07 vs. 7.76 years; p=0.8671) (Table 3). This implies that glycemic control in isolation does not predict the risk of CRS; the damage to the downstream organs - cardiac and renal - does. In their AHA scientific statement on cardiorenal syndrome, Rangaswami *et al.* [6] also reported the same finding, observing that diabetes exacerbates CRS risk by promoting vascular endothelial dysfunction and neurohormonal dysregulation as opposed to hyperglycemia itself. Despite this, the duration-based distribution (Table 4) revealed that CRS-4 was the only type that had cases continuing after over 10 years of T2DM (n=3, 6.67%), indicating that chronic renocardiac pathway may be predisposed in long-term diabetes [2].

## Conclusion:

We show that 33% of T2DM patients had cardiorenal syndrome, which was Type 4, with abnormal renal biomarkers and LVEF reduction as the primary risk factors. Hence, renal evaluation should be done regularly in patients with T2DM. A significant echocardiographic abnormality will help CRS to be identified and treated promptly.

## Figures and Tables

**Table 1:** Distribution of study patients according to type of cardiorenal syndrome (N=45)

**S. No.**	**Type of CRS**	**n**	**%**
1	CRS Type 1	3	6.67
2	CRS Type 2	5	11.11
3	CRS Type 3	0	0
4	CRS Type 4	7	15.56
5	CRS Type 5	2	4.44
6	No CRS	28	62.22
	Total	45	100

**Table 2:** Demographic and clinical characteristics of study patients stratified by CRS Status

**Characteristic**	**CRS Absent (n=28)**	**CRS Present (n=17)**	**p value**
**Sex**			
Male, n (%)	19 (42.22%)	8 (17.78%)	-
Female, n (%)	9 (20.00%)	9 (20.00%)	-
**Age group**			
<50 years, n (%)	6 (13.33%)	4 (8.89%)	-
51-60 years, n (%)	13 (28.89%)	9 (20.00%)	-
61-70 years, n (%)	3 (6.67%)	3 (6.67%)	-
>70 years, n (%)	6 (13.33%)	1 (2.22%)	-
Mean age ± SD (years)	60.29 ± 11.23	56.24 ± 8.24	0.2045

**Table 3:** Comparison of clinical and laboratory variables between CRS-Absent and CRS-Present patients

**Variable**	**CRS Absent (n=28) Mean ± SD**	**CRS Present (n=17) Mean ± SD**	**Mean Difference (95% CI)**	**p value**
Age (years)	60.29 ± 11.23	56.24 ± 8.24	-4.05 (-10.39 to 2.29)	0.2045
Duration of T2DM (years)	8.07 ± 6.25	7.76 ± 5.32	-0.31 (-3.97 to 3.36)	0.8671
Duration of HF (years)	2.52 ± 2.41	1.71 ± 1.30	-0.81 (-2.21 to 0.60)	0.2548
2D Echo LVEF (%)	46.96 ± 7.27	35.18 ± 7.47	-11.79 (-16.34 to -7.23)	<0.0001
HbA1c (%)	8.23 ± 1.36	8.57 ± 1.47	0.34 (-0.53 to 1.20)	0.4371
Serum Urea (mg/dl)	36.86 ± 8.31	60.12 ± 16.61	23.26 (15.76 to 30.75)	<0.0001
eGFR (ml/min/1.73 m^2^)	78.96 ± 15.68	27.47 ± 9.77	-51.49 (-60.03 to -42.95)	<0.0001
Serum Creatinine (mg/dl)	1.13 ± 0.39	2.58 ± 0.91	1.45 (1.05 to 1.84)	<0.0001

**Table 4:** Distribution of CRS Type according to duration of type 2 Diabetes mellitus

**Type of CRS**	**T2DM <5 years n (%)**	**T2DM 5-10 years n (%)**	**T2DM >10 years n (%)**	**Total n (%)**
CRS-1	0 (0.00)	2 (4.44)	1 (2.22)	3 (6.67)
CRS-2	1 (2.22)	4 (8.89)	0 (0.00)	5 (11.11)
CRS-4	1 (2.22)	3 (6.67)	3 (6.67)	7 (15.56)
CRS-5	1 (2.22)	1 (2.22)	0 (0.00)	2 (4.44)
No CRS	8 (17.78)	12 (26.67)	8 (17.78)	28 (62.22)
Total	11 (24.44)	22 (48.89)	12 (26.67)	45 (100.00)

**Table 5:** USG Renal findings and CKD Duration among types of cardio renal syndrome

Type of CRS	CMD Altered n (%)	CMD Lost n (%)	No USG Change n (%)	CKD 1-3 yrs n (%)	CKD 3-5 yrs n (%)	CKD >6 yrs n (%)	No CKD n (%)	Total n (%)
CRS-1	0 (0.00)	0 (0.00)	3 (6.67)	0 (0.00)	0 (0.00)	0 (0.00)	3 (6.67)	3 (6.67)
CRS-2	4 (8.89)	0 (0.00)	1 (2.22)	0 (0.00)	0 (0.00)	0 (0.00)	5 (11.11)	5 (11.11)
CRS-4	0 (0.00)	7 (15.56)	0 (0.00)	2 (4.44)	4 (8.89)	1 (2.22)	0 (0.00)	7 (15.56)
CRS-5	0 (0.00)	0 (0.00)	2 (4.44)	0 (0.00)	0 (0.00)	0 (0.00)	2 (4.44)	2 (4.44)
No CRS	1 (2.22)	0 (0.00)	27 (60.00)	0 (0.00)	0 (0.00)	0 (0.00)	28 (62.22)	28 (62.22)
Total	5 (11.11)	7 (15.56)	33 (73.33)	2 (4.44)	4 (8.89)	1 (2.22)	38 (84.44)	45 (100.00)
